# Perifascial Nodular Fasciitis Adjacent to the Tensor Fasciae Latae: Ultrasound and MRI Findings in a Rare Hip Presentation

**DOI:** 10.7759/cureus.102906

**Published:** 2026-02-03

**Authors:** Amine Benfaida, El Mehdi Mniai, Amine Belokda, Anas Rguig-Assakali, Amal Rami

**Affiliations:** 1 Radiology, Cheikh Khalifa International University Hospital, Mohammed VI University of Health Sciences, Casablanca, MAR; 2 Radiology, Mohammed VI Center for Research and Innovation, Cheikh Khalifa International University Hospital, Mohammed VI University of Health Sciences, Casablanca, MAR

**Keywords:** differential diagnosis, hip, mri, nodular fasciitis, soft tissue tumor, tensor fasciae latae, ultrasound

## Abstract

Nodular fasciitis is a benign, self-limited myofibroblastic proliferation that can mimic soft-tissue sarcoma because of rapid growth. It most often affects the upper extremities, while presentation around the hip remains uncommon. We report a 29-year-old woman who noticed a recently appearing, painless lateral hip mass that prompted medical consultation. Ultrasound and magnetic resonance imaging helped localize and characterize the lesion and guided biopsy. Histology confirmed nodular fasciitis. This case highlights the value of imaging-pathology correlation in recognizing this benign entity and avoiding unnecessary aggressive treatment.

## Introduction

Nodular fasciitis is a benign, self-limited myofibroblastic proliferation that grows quickly and often adopts a pseudosarcomatous pattern. Clinicians and radiologists often suspect malignancy because the lesion appears over a short time, shows high cellularity on pathology, and can display striking imaging features. Nodular fasciitis represents about 0.025% of all soft-tissue tumors and accounts for roughly 10-11% of benign fibroblastic lesions [1,2].

Nodular fasciitis most often affects young adults in the second to fourth decades, with no strong sex predilection [3]. It arises most frequently in the upper extremities (≈45-50%), followed by the trunk (≈20-30%) and the head and neck region (≈15-20%) [2,4]. Reports involving the lower limb remain uncommon (<15%), and cases occurring along the tensor fasciae latae region appear only sporadically in the literature [5-7].

Imaging guides the diagnostic pathway because nodular fasciitis can resemble malignant soft-tissue tumors, especially myxoid sarcomas and fibromatosis. Ultrasound localizes the lesion and supports image-guided sampling, while MRI refines tissue characterization and maps local extent. We report a rare case of nodular fasciitis arising along the tensor fasciae latae region and emphasize how ultrasound-MRI correlation, paired with radiologic-pathologic concordance, supports accurate diagnosis and avoids unnecessary aggressive management.

## Case presentation

A 29-year-old woman with no prior medical or surgical history noticed a recently appearing, progressively enlarging, painless palpable swelling over the lateral aspect of her left hip, which prompted medical consultation. She reported no trauma, fever, weight loss, or other systemic symptoms and denied functional limitation or neurological complaints.

On physical examination, we palpated a firm, superficial nodule in the anterolateral region of the left hip. The lesion appeared poorly mobile relative to deeper planes, without overlying skin changes, local warmth, or tenderness. Hip range of motion remained preserved and symmetrical. No regional lymphadenopathy was identified. Routine laboratory tests, including inflammatory markers, were within normal limits.

**Figure 1:**
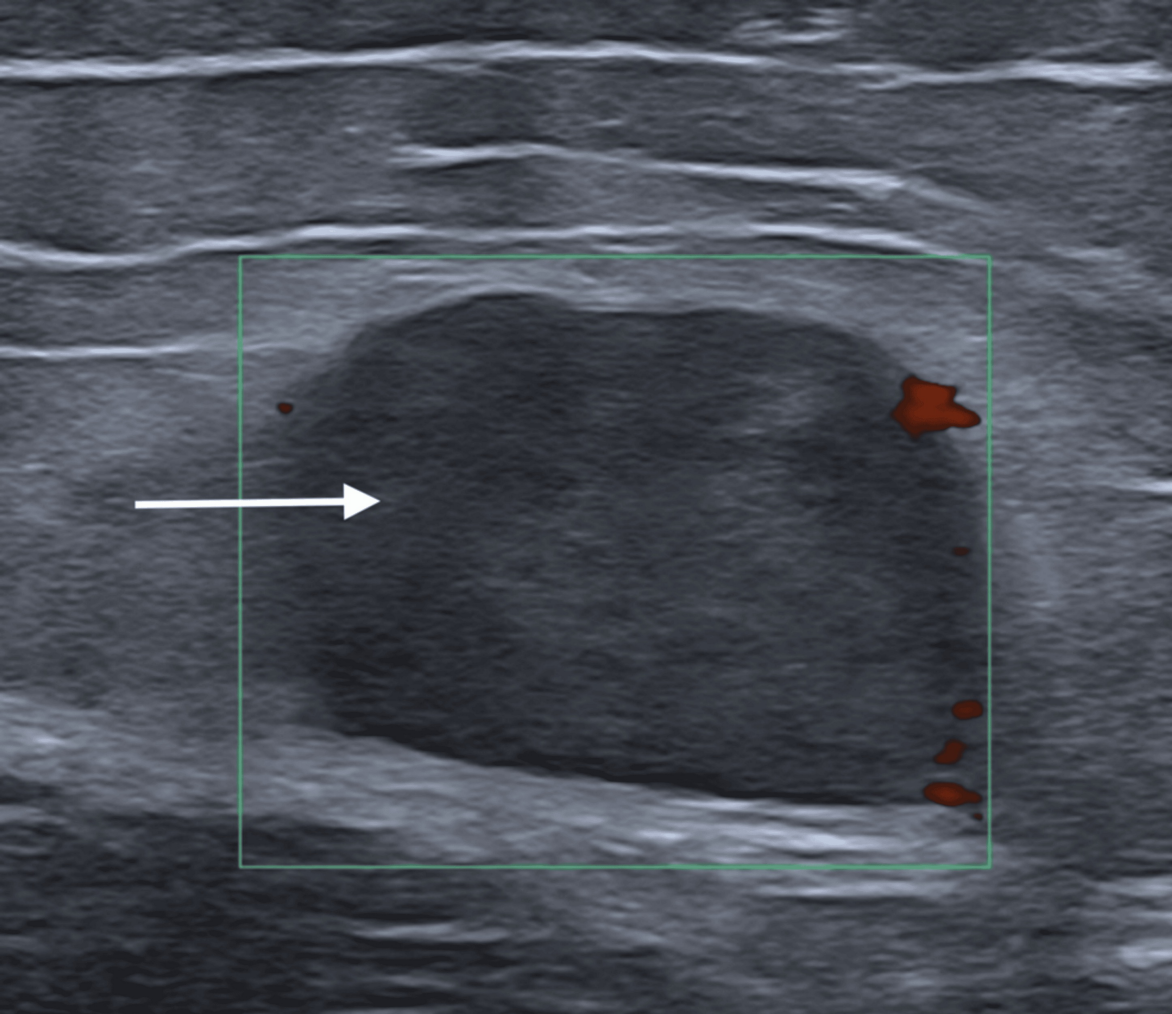
Ultrasound of the left hip shows a well-defined, oval, hypoechoic subcutaneous soft-tissue mass overlying and adjacent to the tensor fasciae latae (white arrow). Color Doppler shows no meaningful internal vascularity.

Ultrasound demonstrated a well-defined superficial soft-tissue nodule along the fascia overlying the left tensor fasciae latae. The lesion appeared hypoechoic with heterogeneous internal echotexture and showed no significant vascularity on color Doppler imaging. Surrounding tissues remained preserved, with no fluid collection or calcification (Figure 1).

**Figure 2:**
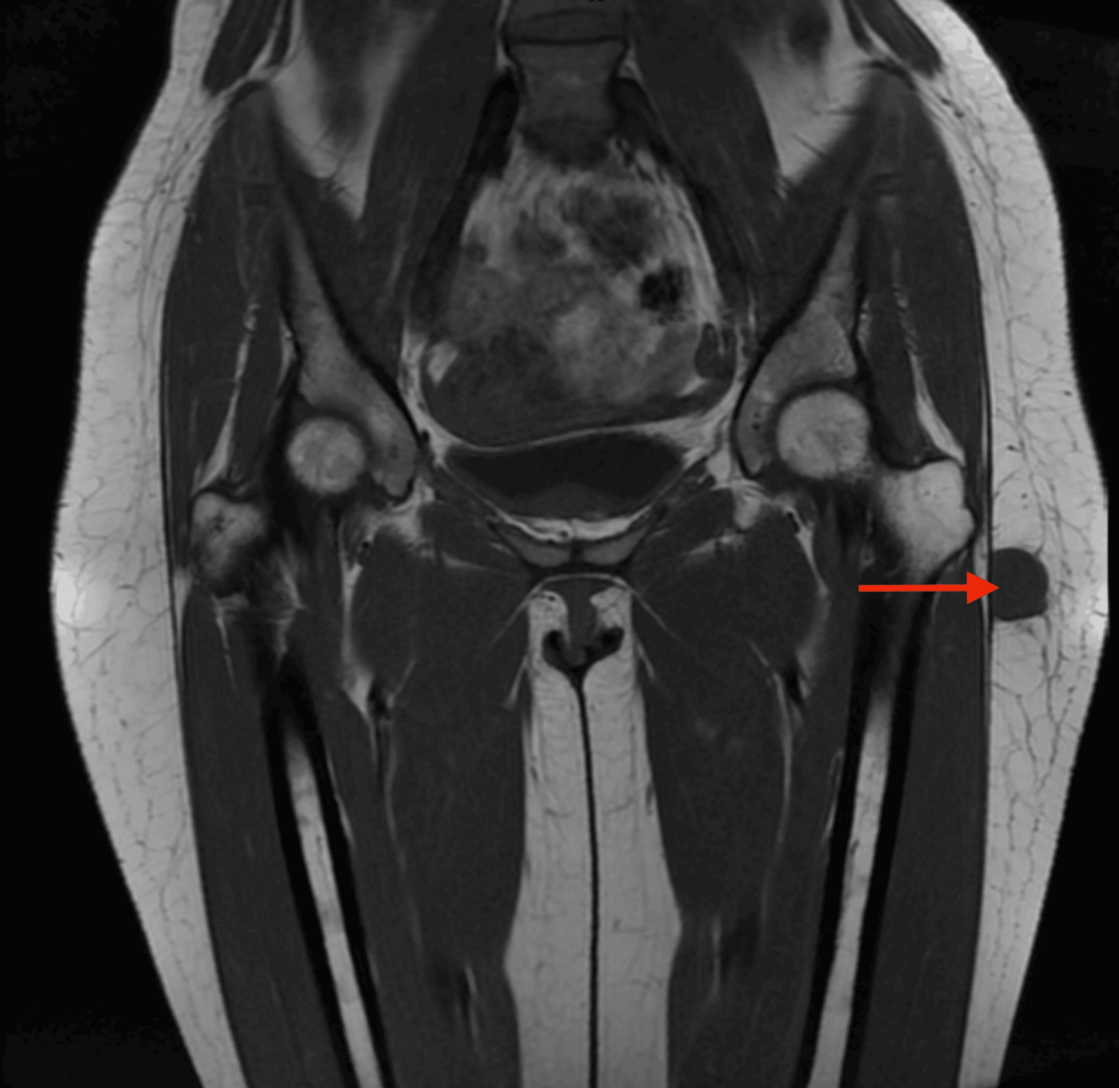
Coronal T1-weighted MRI of the pelvis shows a well-circumscribed nodular lesion in the superficial soft tissues along the lateral aspect of the left hip, adjacent to the tensor fasciae latae (red arrow). The lesion is isointense to skeletal muscle, with no adjacent bone involvement.

**Figure 3:**
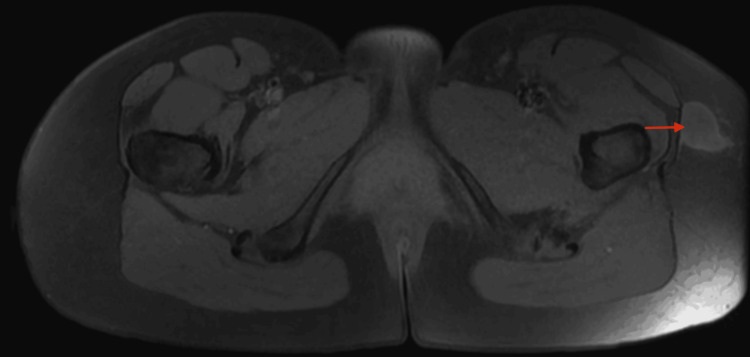
Axial non–contrast-enhanced T1-weighted fat-suppressed pelvic MRI shows a well-circumscribed superficial soft-tissue nodule along the lateral aspect of the left hip, adjacent to the tensor fasciae latae (red arrow). The lesion remains isointense to skeletal muscle and shows no intralesional fat, with preserved surrounding planes and no adjacent bone involvement.

**Figure 4:**
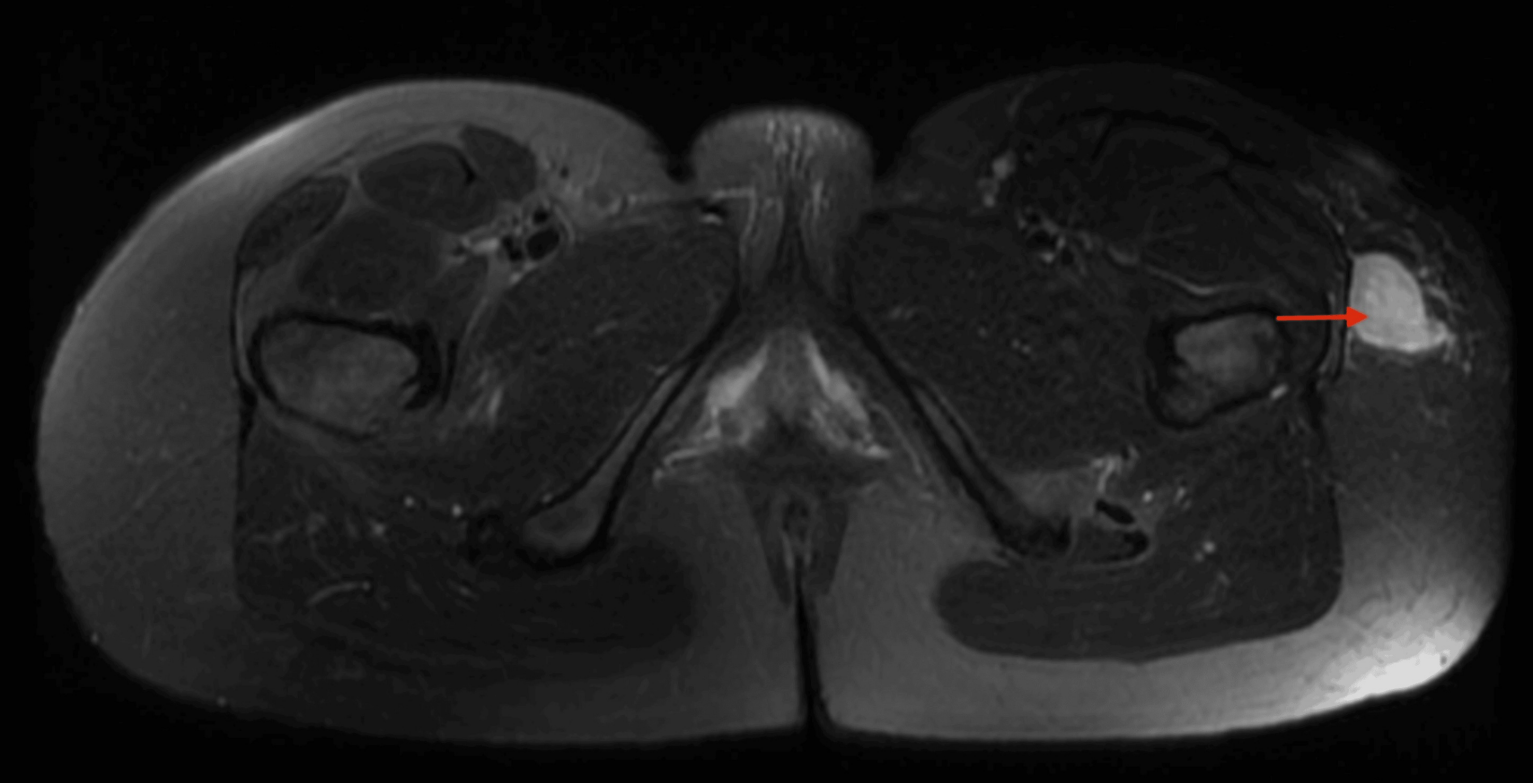
Axial T2-weighted fat-suppressed pelvic MRI shows a well-circumscribed, heterogeneously hyperintense superficial nodule along the lateral aspect of the left hip, adjacent to the tensor fasciae latae (red arrow). The surrounding fat planes remain preserved, with no deep fascial breach or extension into the underlying muscle.

**Figure 5:**
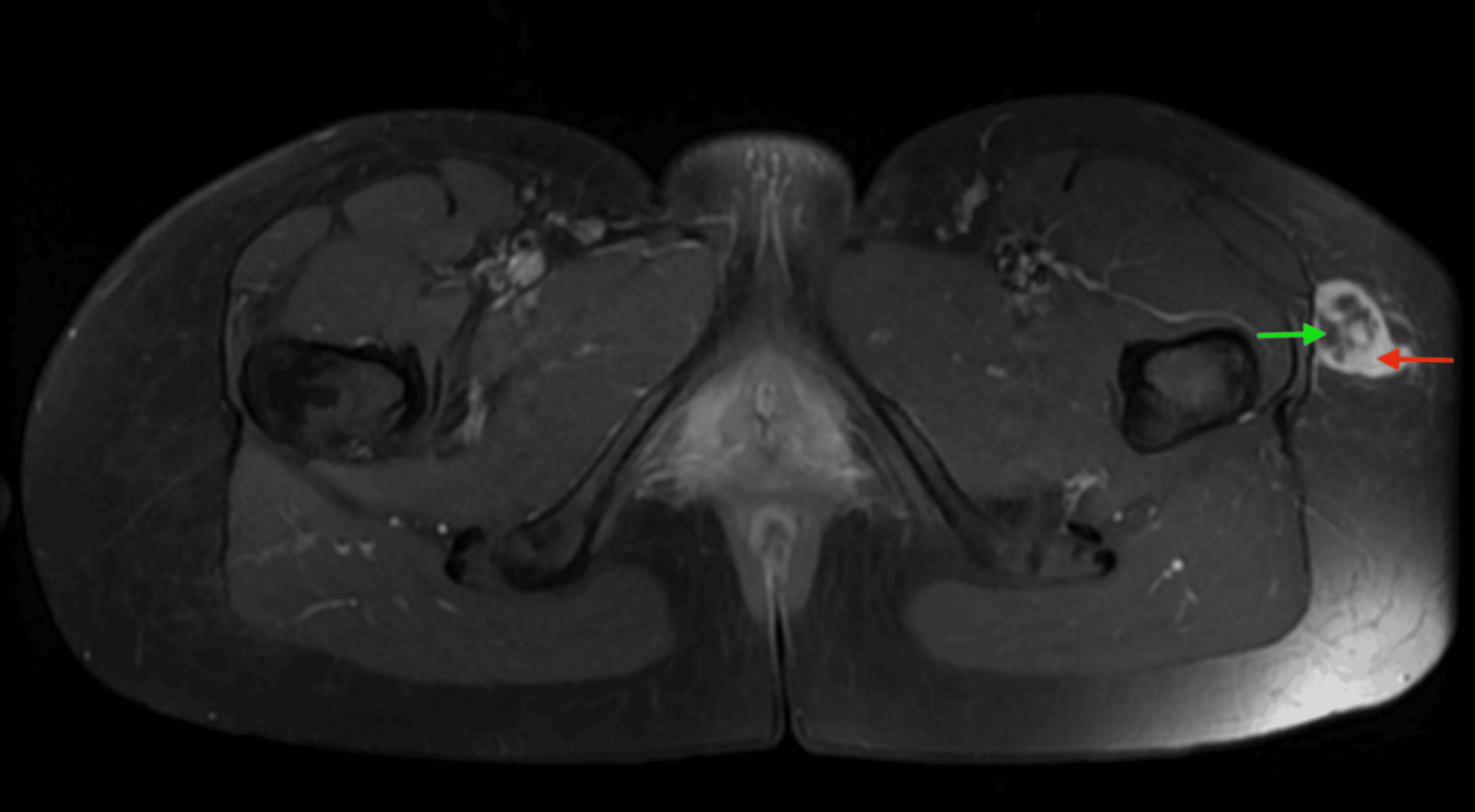
Axial contrast-enhanced T1-weighted fat-suppressed pelvic MRI shows a well-circumscribed superficial nodule along the lateral aspect of the left hip, adjacent to the tensor fasciae latae (red arrow), with heterogeneous peripheral enhancement and a central non-enhancing area, consistent with a hypocellular or myxoid stromal component (green arrow). No deep fascial breach, bone involvement, or neurovascular encasement is present.

MRI of the pelvis further characterized the lesion. Coronal T1-weighted images demonstrated a well-circumscribed, oval nodular lesion developing along the tensor fasciae latae fascia, in close contact with the muscle surface, measuring approximately 24 × 20 mm in the axial plane and extending over 25 mm craniocaudally, with signal intensity isointense to skeletal muscle. Axial non-contrast T1-weighted fat-suppressed images showed no signal dropout, which argued against intralesional fat or hemorrhagic components (Figures 2, 3). T2-weighted fat-suppressed and short TI inversion recovery (STIR) sequences demonstrated heterogeneous hyperintensity with subtle internal septations, without surrounding edema or disruption of the aponeurotic plane (Figure 4). Post-contrast T1-weighted fat-suppressed images showed heterogeneous peripheral enhancement with a central non-enhancing area; given the absence of aggressive features, this pattern favored a hypocellular/myxoid stromal portion rather than necrosis. No bone involvement or neurovascular encasement was identified (Figure 5).

Based on the clinical presentation and imaging findings, the differential diagnosis included nodular fasciitis, a myxoid soft-tissue tumor, and, less likely, a low-grade sarcoma. An ultrasound-guided core needle biopsy was performed.

Histologic analysis demonstrated a proliferation of spindle-shaped myofibroblasts arranged in short fascicles within a myxoid to collagenous stroma, associated with extravasated erythrocytes and a mild inflammatory infiltrate. No significant cytologic atypia or atypical mitotic figures were identified. These findings supported the diagnosis of nodular fasciitis. (The original histopathological slides from the initial biopsy could not be retrieved from the referring institution and were therefore unavailable for review.)

Given the benign diagnosis and the absence of symptoms or functional impairment, conservative management with clinical and imaging follow-up was adopted. Subsequent follow-up demonstrated partial spontaneous regression of the lesion, and the patient remained asymptomatic.

## Discussion

Nodular fasciitis poses a recurrent diagnostic dilemma because it combines rapid growth, high cellularity, and variable imaging features that can resemble malignant soft-tissue tumors. This pseudosarcomatous behavior often raises concern for sarcoma and may drive overly aggressive workup or treatment when clinicians do not recognize the entity.

Epidemiologically, nodular fasciitis predominates in young adults, with roughly 70% of cases occurring before 40 years of age and no consistent sex predilection [3]. The upper extremities represent the most frequent site, whereas the lower limb accounts for fewer than 15% of reported cases [4]. Lesions arising along the lateral hip fascia have rarely been reported, and reports describing perifascial nodular fasciitis adjacent to the tensor fasciae latae remain scarce [6-8]. This unusual anatomic setting increases diagnostic uncertainty and highlights the value of careful imaging assessment.

Clinically, patients usually notice a rapidly appearing mass developing over weeks. Pain varies and may remain absent, as in our patient. Preserved joint mobility and the absence of systemic symptoms or neurological deficit may suggest a benign process, yet imaging and histology still guide final decision-making.

Imaging, therefore, plays a central role. Ultrasound typically shows a well-defined hypoechoic or heterogeneous soft-tissue lesion, with Doppler vascularity ranging from absent to moderate, depending on lesion cellularity and timing [5,7]. In addition to lesion detection, ultrasound clarifies the relationship to fascia and adjacent muscle and provides a straightforward route for image-guided biopsy. MRI refines tissue characterization and local extent. Typical features include a T1 signal isointense to muscle and heterogeneous hyperintensity on T2-weighted or fat-suppressed sequences, which reflect the variable proportions of cellular and myxoid components [8,9].

Enhancement patterns also vary. Diffuse or peripheral enhancement remains common, while a central non-enhancing area, such as in our case, may raise the question of necrosis. In nodular fasciitis, this appearance more often reflects hypocellular or myxoid stromal portions rather than true necrosis, particularly when surrounding edema, aggressive margins, or bone involvement remain absent. Correlating enhancement with T2-weighted signal and the clinical course helps support this interpretation.

Even with advanced imaging, overlap persists with sarcoma, fibromatosis, and other myxoid tumors. Histologic confirmation, therefore, remains essential, especially for deep-seated or atypically located lesions. The characteristic microscopic pattern of spindle-shaped myofibroblasts arranged in short fascicles within a myxoid to collagenous stroma, often with extravasated erythrocytes, supports a confident diagnosis and helps avoid overtreatment.

Most cases follow a benign, self-limited course. Studies have reported partial or complete spontaneous regression in up to 10-20% of patients [2]. Clinicians usually reserve excision for persistent symptoms, functional impairment, or unresolved diagnostic uncertainty. In our patient, radiologic-pathologic concordance supported conservative management, with a favorable outcome that illustrates the practical value of a multidisciplinary approach.

## Conclusions

Nodular fasciitis presenting along the lateral hip fascia adjacent to the tensor fasciae latae remains exceptionally uncommon and may raise concern for malignancy because of its rapid growth and sometimes atypical imaging appearance. While ultrasound and MRI help localize the lesion and assess its extent, histology secures the diagnosis. Clinicians can avoid misdiagnosis and unnecessary aggressive management by recognizing this entity and maintaining close radiologic-pathologic correlation. Once pathology confirms the benign nature of the lesion, conservative follow-up offers a reasonable and effective option.
